# Mean-field theory of social laser

**DOI:** 10.1038/s41598-022-12327-w

**Published:** 2022-05-20

**Authors:** Alexander P. Alodjants, A. Yu. Bazhenov, A. Yu. Khrennikov, A. V. Bukhanovsky

**Affiliations:** 1 ITMO University, Kronverksky Av. 49, bldg. A, St. Petersburg, 197101 Russia; 2 International Center for Mathematical Modeling in Physics, Engineering, Economics, and Cognitive Science Linnaeus University, Vaxjo-Kalmar, 35195 Sweden

**Keywords:** Mathematics and computing, Biophysics

## Abstract

In this work we suggest a novel paradigm of social laser (solaser), which can explain such Internet inspired social phenomena as echo chambers, reinforcement and growth of information cascades, enhancement of social actions under strong mass media operation. The solaser is based on a well-known in quantum physics laser model of coherent amplification of the optical field. Social networks are at the core of the solaser model; we define them by means of a network model possessing power–law degree distribution. In the solaser the network environment plays the same role as the gain medium has in a physical laser device. We consider social atoms as decision making agents (humans or even chat bots), which possess two (mental) states and occupy the nodes of a network. The solaser establishes communication between the agents as absorption and spontaneous or stimulated emission of socially actual information within echo chambers, which mimic an optical resonator of a convenient (physical) laser. We have demonstrated that social lasing represents the second order nonequilibrium phase transition, which evokes the release of coherent socially stimulated information field represented with the order parameter. The solaser implies the formation of macroscopic social polarization and results in a huge social impact, which is realized by viral information cascades occurring in the presence of population imbalance (social bias). We have shown that decision making agents follow an adiabatically time dependent mass media pump, which acts in the network community reproducing various reliable scenarios for information cascade evolution. We have also shown that in contrast to physical lasers, due to node degree peculiarities, the coupling strength of decision making agents with the network may be enhanced $\sqrt{\langle k\rangle }$ times. It leads to a large increase of speed, at which a viral message spreads through a social media. In this case, the mass media pump supports additional reinforcement and acceleration of cascade growth. We have revealed that the solaser model in some approximations possesses clear links with familiar Ising and SIS (susceptible-infected-susceptible) models typically used for evaluating a social impact and information growth, respectively. However, the solaser paradigm can serve as a new platform for modelling temporal social events, which originate from “microscopic” (quantum-like) processes occurring in the society. Our findings open new perspectives for interdisciplinary studies of distributed intelligence agents behavior associated with information exchange and social impact.

## Introduction

The current life-style may be characterized as a sequence of on-line representations in various networks. The rapid growth of information (network) resources in terms of information exchange and processing leads to an exponential growth in the information being processed. Sometimes, enormous (social) enhancement effect can result from unimportant, at first sight, information spread. Contrary, in some cases, socially actual information and knowledge rapidly attenuate. How fast does socially actual (s)-information spread in social networks and communities and how are these communities transformed under some perturbations? These questions are becoming critical for a well-functioning society. To find answers to these questions we suggest a novel paradigm of social laser, shortly solaser, which is based on a well-known in quantum physics laser model of coherent amplification of an optical field. The model may be explored to describe real life socially actual information processing and reinforcement in the presence of strong mass media action, which is determined as a mass media pump.

Lasers represent one of keystone achievements obtained in quantum theory and experiment within more than 100 years^1,2^. Current quantum technologies provide designing of various laser-like quantum devices, which produce photon^3,4^ or coherent matter states^5^. At the heart of these devices, we can infer seminal laser principles of bosonic stimulation and amplification. These principles were laid back in the 60s of the last century by the fathers of modern laser science^6–8^.

Understanding the physics of laser generation and coherence has always been a key task for laser physics. Main (coherent) statistical features of laser irradiation were explained by examining photon second order correlation function within quantum and nonlinear optics theory^9–13^. Another theory of phase transitions and critical phenomena based on statistical and thermodynamic approach was able to explain coherent phase ordering in condensed matter^14^, and macroscopic coherent effects of superconductivity^15^.

The relationship between these two theories has always been the subject of heated debates that beneficially affected laser science as a whole. The main difference between the two fundamental theoretical approaches is that initially phase transitions were considered only for thermodynamic equilibrium systems^15^. However, laser systems are not thermodynamically equilibrium. Physically, it is more suitable to discuss some analogies occurring in open (driven-dissipative) and thermodynamically equilibrium laser-like systems exhibiting phase transitions^16–18^. Current status of various approaches applied to studies of phase transitions occurring in quantum driven-dissipative open systems it is possible to find in^18^.

Nowadays, the role of thermodynamic equilibrium in the photon gas coherence is under discussion^19–24^. The interaction of these theories and relevant discussions has reached a qualitatively new level, cf.^25–30^.

With the development of quantum technologies in practice, scientists were able to experimentally observe a Bose-Einstein condensate (BEC), a new macroscopic coherent state of matter. It was found for atoms^31^, exciton-polaritons^32^, photons^33^, magnons^34^, and phonons^35^. Remarkable achievements in this area are also due to significant progress in manipulation and trapping of quantized light field, which represents an indispensable tool for current cavity QED technologies, cf.^36,37^. To some extend, the application of various theoretical approaches to study the systems exhibiting BEC and/or lasing phenomena is a matter of convenience for the system description. In real life systems are not usually in thermal equilibrium due to their open (driven-dissipative) nature, and finite size effects should also be considered. Sometimes it is possible to examine system within short time scales when influence of various dissipative processes may be neglected. In this case we are able to use thermodynamic approach. In particular, such terms as “atom laser” and “polariton laser”, which are actually not lasers in their original sense and denote the coherent (ordered) state of matter, have been established for atomic and polariton condensates, respectively. Moreover, exciton-polariton condensates are also not in thermodynamic equilibrium. However, trapped in microcavity exciton-polaritons clearly exhibit phase transition to condensation in a strong matter-field coupling regime^32^.

The examples discussed above relate to condensed or solid state systems, which are ordered in a certain way, or not ordered at all, like gases. The study of phase transitions in complex structured systems has recently been of great interest^38–40^. Although systems, such as spin glasses, have been studied for a long time, these are various complex networks that are in the focus now^41^. The main reason for the growing interest in such systems is the large-scale Internet network development and digitalization, which cover all aspects of humans life^42^. Providing subscribers with an opportunity to socialize globally, the Internet itself has become a social phenomenon that unites people in various social communities and on business and educational platforms. By examining phase transitions in such networks, we are able to make some conclusions (within certain limits) about social, information, communication, and emotional processes occurring in cyberspace^43–45^. In particular, public (social) networks promote a coherent macroscopic social impact involving a large number of people on-line. They provide information amplification and cascading, which may be elucidated by computer-mediated social studies^46^. In real life, coherent processes occurring in the networks provide social cohesion, which in some cases leads to cascading social processes (Arab spring, Occupy Wall Street movements, etc. ) in different countries^47–49^.

One of intriguing problems at the nexus of modern quantum physics and statistical physics, social sciences, cognitive and computer-oriented studies is how close various statistical (laser-like) physical models may describe “on-line” social events occurring due to current Internet communication facilities. From physical point of view the coherent processes discussed are similar to laser field generation, which results in huge energy release in ensemble of two- (or multi-) level oscillators under population inversion conditions. Andrei Khrennikov was the first who defined the social laser revealing the analogy between the formation of laser irradiation and real life social actions. He regarded coherent social processes as laser-like actions of a new type, cf.^50,51^. This deep analogy manifests itself at different stages of laser irradiation formation^52^. For example, the use of a resonator in a convenient laser is similar to the social effect of echo chambers, which has been experimentally obtained within social networks, cf.^53–55^. In this regard, the social laser paradigm, based on the use of socially-oriented networks as laser “medium”, significantly differs from the ideas of exploring laser-like models outside physics, established many years ago, see^56^, cf.^57^. Since the laser system is an open system, it can attract more people at each act of social laser stimulation.

Notably, current studies of processes occurring in social communities are based on fundamentals of statistical physics and phase transitions theory. Traditionally, the approach of statistical physics is applied to complex systems description, which can include various social networks exhibiting social communities, cf.^58^. In particular, statistical Ising model is at the basis of many socially oriented statistical models, which are related to the exchange of information between people^40,59–62^. A simplified two-level (spin-like) decision making (DM) agents model may be used in this case; DM agents represent so-called social atoms^63^. In particular, Ising-like models suppose collective opinion formation and social impact^64,65^, epidemic, information and rumor spreading^66–68^. However, the major part of the models examined is thermodynamically equilibrium, whereas real-life social processes require a non-equilibrium approach. In this sense, the problem of non-equilibrium phase transition is of primary interest, as it may appear in laser-like systems possessing Ising-like Hamiltonians, cf.^18,69^. Such models become crucial for feature modelling of distributed intelligence systems, which presume artificial and natural intelligence agents of DM behaviour and their interaction capacity in the framework of a socially oriented network^70^.

This work aims to establish a simple (generic) model of a solaser as a system possessing the second order (non-equilibrium) phase transition, which presumes a complex network topology inherent to real network social communities. To be more specific, we examine networks, which may be characterized by means of power law degree distribution function. Such networks allow for a relative simple analytical description^44^. Here it is the good place to mention the quantum field approach to modeling of cognition and consciousness (Vitiello^71,72^) explaining the long range correlations in the brain.

## Results

### Equilibrium phase transition in Ising model on complex networks

#### Network models with Ising-type interaction

**Figure 1:**
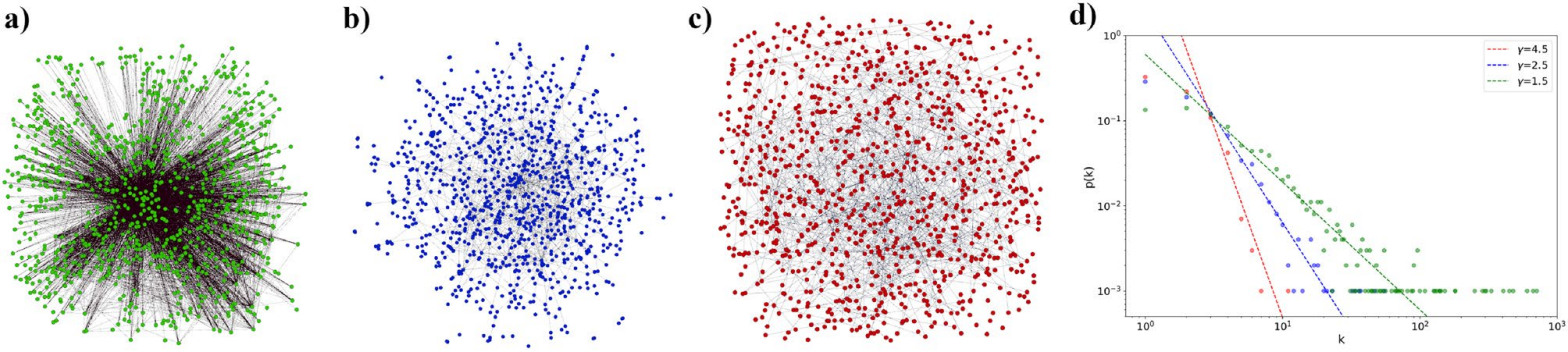
Power-law degree distribution networks for (**a**) $\gamma =1.5$, (**b**) $\gamma =2.5$ and (**c**) $\gamma =4.5$, which correspond to anomalous, scale-free and random regimes, respectively, (**d**) power–law degree distributions in a logarithmic scale for the networks given in (**a**–**c**). The number of nodes is $N=1000$.

We start with the Ising model defined on annealed networks. We assume that two-level (spin-1/2) quantum systems randomly occupy *N* nodes of a complex network—see Fig. 1. In the framework of social community analysis we model DM agents with these simple quantum systems and assume that they possess information exchange action. Thus, we represent this community as a graph with non-trivial (specific) properties. The Ising Hamiltonian of the model reads as

1 $$\begin{aligned} \text {H}=-\sum \limits _{ij} {{J_{ij}}\sigma ^{z}_i\sigma ^{z}_j}-\frac{1}{2}\sum \limits _{i} {h_i\sigma ^{z}_i}, \end{aligned}$$

where $\sigma ^{z}_i$, $i=1,...,N$, characterize the *i*-th agent DM spin component.

The first term in (1) characterizes interaction between DM agents. In real life such an interaction is implemented by some information exchange, which is still hidden for an external “observer” within the Ising model. Notably, for this model we cannot predict directly how information is relevant to DM agents inhomogeneously distributed over the network. However, we can conclude how this information spreads within the network because of the first term in (1), which depends on the network topology. The sum in (1) is performed over the graph vertices with certain adjacency matrix $A_{ij}$ proportional to $J_{ij}$, which stores the information about the graph structure: matrix element $A_{ij}=1$ if two vertices are linked and $A_{ij}=0$ otherwise.

Parameter $h_i$ in (1) characterizes the coupling of a DM agent with external (information) field. We examine the case when this information is the same for all DM agents and represents classical (strong) information pumping. In other words, we set $h_i=h$ for arbitrary *i* (hereafter for simplicity we put the Planck and Boltzmann constants $\hbar =1$, $k_B=1$).

We are interested in the annealed network approach that presumes a weighted, fully connected graph model. The network dynamically rewires. We recast parameter $J_{ij}$ that indicates the coupling between the nodes in Eq. (1) through probability $p_{ij}$ as $J_{ij}=Jp_{ij}$, where *J* is a constant, and $p_{ij}$ is the probability for two nodes *i* and *j* to be connected:

2 $$\begin{aligned} p_{ij}=P(A_{ij}=1)=\frac{k_i k_j}{{N\langle }k\rangle }, \end{aligned}$$

where $A_{ij}$ is an element of the adjacency matrix, $k_i$ is *i*-th node degree, which indicates an expected number of the node neighbors and is taken from distribution *p*(*k*). In (2) $\langle {k}\rangle ={\frac{1}{N}}\sum \limits _{i}{k_i}$ is an average degree. Noteworthy, the annealed network approach is valid for $p_{ij}\ll 1$ and large enough *N*, cf.^44^. Thus, the strength of two spins interaction $J_{ij}$ is a variable parameter and depends on particular network characteristics; it is greater for two pairs of nodes with the highest *k* coefficient.

In practice, different approaches provide an explanation for a real world network topology^40^. Such networks may exhibit the power-law degree distribution, cf.^73^. Since the number of nodes is large enough, $N\gg 1$, we are interested in network structures, which admit continuous degree distribution *p*(*k*). To be more specific, in this work we examine networks with distribution function *p*(*k*) defined as

3 $$\begin{aligned} p(k)=\frac{(\gamma -1)k_{min}^{\gamma -1}}{k^{\gamma }}, \end{aligned}$$

where $\gamma$ is a degree exponent that covers anomalous ($1<\gamma <2$)—Fig. 1a, scale-free ($2<\gamma <3$)—Fig. 1b, and random ($\gamma >3$)—Fig. 1c regimes, cf.^44^. The properties of scale-free networks possessing distribution (3) for $\gamma =2$ and $\gamma =3$ should be calculated separately.

The normalization condition for *p*(*k*) is represented as

4 $$\begin{aligned} \int \limits ^{+\infty }_{k_{min}}{p(k)dk}=1. \end{aligned}$$

The scale-free networks possess preferential attachment phenomena, which result in hubs appearance. The largest hub is described by degree $k_{max}$ called a natural cutoff. The condition

5 $$\begin{aligned} \int \limits ^{+\infty }_{k_{max}}{p(k)dk}=\frac{1}{N} \end{aligned}$$

can be used if the network with *N* nodes possesses more than one node with $k>k_{max}$.

From (4), (5) we immediately obtain

6 $$\begin{aligned} \begin{aligned} k_{max}=k_{min}N^{\frac{1}{\gamma -1}}. \end{aligned} \end{aligned}$$

Figure 1d demonstrates probability distribution function (3) plotted in a logarithm scale for the networks shown in Fig. 1a–c. The node degree fluctuations grow at $\gamma \le 3$, cf.^69^. Hubs in Fig. 1d appear as dots in the right corner of the distribution function. The number of hubs and their size dramatically grow with vanishing $\gamma$ in the anomalous regime where $k_{max}/k_{min}>N$, cf. Fig. 1a and the green line in Fig. 1d.

Real-world networks mostly possess degree exponent $\gamma >2$. The Bianconi-Barabási (BB) network appropriately models such a network. In particular, the BB network is based on a fitness model, which accounts that the probability of the newly attached node link is proportional to its fitness parameter^44^. This parameter may be interpreted in the framework of statistical mechanics approach and leads to $\gamma = 2.255$^38^. In particular, it is possible to map the BB network to gas particles assuming that energy of each node is determined by its fitness parameter. As a result, we can clearly distinguish the new BEC phase of the network. The network condensation phenomena presumes super-hub formation, which accumulates a huge number of links; strictly speaking, such a network is not a scale-free any more.

The statistical properties of networks may be characterized by means of the *n*-th moment for the degree distribution defined as:

7 $$\begin{aligned} K^{(n)}\equiv {{\langle }k^n{\rangle }}=\int \limits ^{k_{max}}_{k_{min}}{{k^n}p(k)dk}, \end{aligned}$$

where *n* is a positive integer. In this work, we are interested in the first and normalized *n*th order ($n=2,3,4$) degree correlation functions, which are defined as

8 $$\begin{aligned} \zeta _{n}\equiv \frac{K^{(n)}}{K^{(1)}}, \quad n=2,3,4. \end{aligned}$$

**Figure 2:**
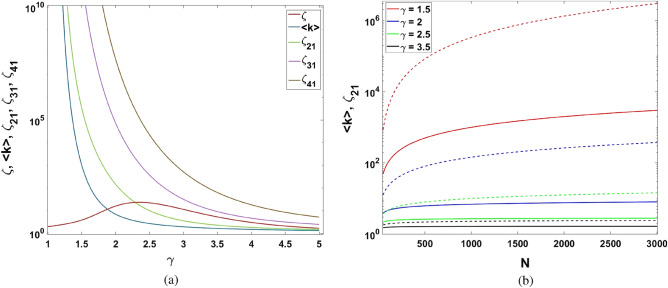
Dependence of (**a**) $\zeta$, ${\langle }k\rangle$, $\zeta _{21}\equiv\zeta _{2}$, $\zeta_{31}\equiv\zeta _{3}$ and $\zeta_{41}\equiv\zeta _{4}$ on degree exponent $\gamma$ for $k_{min}=1$, $N=1000$, and (**b**) $\zeta _{21}$ (dash line) and ${\langle }k\rangle$ (solid lines) on *N* for $k_{min}=1$ and different values of $\gamma$. Both figures are plotted in logarithmic scale.

In Fig. 2 we represent the main properties of scale-free network statistical characteristics as a function of degree exponent $\gamma$.

Remarkably, the *n*th order correlation functions defined in (7) diverge at $\gamma =1$. On the contrary, their combination $\zeta \equiv [K^{(1)}]^2 K^{(4)}/[K^{(2)}]^3$ remains finite.

#### Network topology stimulating phase transition

Here, we use the Hamiltonian formalism, which is common in physics and widely used in the framework of DM agents interaction, see e.g.^40,61,62,64,65^. It allows to speak about some social energy stored in  (1). In this sense, the interpretation of social spin is straightforward: different spin components correspond to agents preferences with the same spin energy. For example, with no external information in elections people as DM agents can prefer to vote in a candidate or reject them without any social energy changing. The external (information) field or interaction with other agents influences our potential decisions. External information may be enough to flip our decision or not. In this case, the temperature, *T*, represents some vital macroscopic parameter, which manifests about microscopic processes occurring in the system .

In physics temperature indicates energy exchange for the canonical ensemble of fixed number of particles with the thermal bath. In social science it is also possible to introduce social temperature; it stands for some sophisticated (fitting) parameter, which may be discussed in the framework of various models in economy, finance, etc., cf.^74–76^. In this work, we assume that social temperature indicates abilities of DM agents for information exchange with other agents and with an environment, respectively. In other words, thermalization, which is a key-stone problem for any physical system, may be achieved in social networks through information exchange. In this case, the system approaches thermal equilibrium at time periods long enough. Comparing our model with the models, which presume money exchange financial markets, we can identify the temperature parameter as an average number of messages per agent in the network, cf.^74,75^.

In this work we consider the mean-field approach to the system described by the Ising Hamiltonian (1). At social level of DM, the system is totally characterized by the order parameter $S_z$ defined as

9 $$\begin{aligned} S_z=\frac{1}{N{{\langle }k{\rangle }}}\sum \limits _{i}k_i\langle {\sigma ^{z}_i}\rangle , \end{aligned}$$

and represents a weighted average spin component, cf.^56^. Collective variable $S_z$ obeys self-consistent equation

10 $$\begin{aligned} S_z=\frac{1}{\langle {k}\rangle }\int \limits ^{k_{max}}_{k_{min}}{kp(k){\text {tanh}\left[ \frac{{\beta }}{2}(4J S_z k+h)\right] }}dk, \end{aligned}$$

where $\beta \equiv 1/T$ is reciprocal temperature; in (10) substitution $\frac{1}{N}\sum \limits _{i}{...} \rightarrow \int \limits ^{k_{max}}_{k_{min}}{...p(k)dk}$ is performed.

At high enough temperatures and external field $h=0$ Eq. (10) admits non-zero solution for collective spin component $S_z$. This solution corresponds to some ferromagnetic (FM) phase possessing $S_z\ne 0$ and indicating some certain DM at macroscopic level. Critical reciprocal temperature $\beta _c$, which provides this solution, is determined from Eq. (10) and represented as

11 $$\begin{aligned} \beta _c=\frac{1}{2J\zeta _{2}}, \end{aligned}$$

where parameter $\zeta _{2}$ defined in Eq. (8).

The dependence given in (11) admits simple interpretation in the framework of information exchange. In particular, the strongest interaction between the DM agents evokes the highest critical temperature, $T_c\equiv 1/\beta _c$. Simultaneously, a large number of hubs can manifest the activity of DM agents. In this limit $\zeta _{2}$ is also large enough and corresponds to high temperature of phase transition $T_c$. Critical temperature $T_c$ becomes very large ($\beta _c\simeq 0$) in the vicinity of $\gamma =1$ where $\zeta _{2}$ enormously increases, see Fig. 2a. In the opposite case, for large degree exponent $\gamma$ the $\zeta _{2}$ approaches $k_{min}$ and critical temperature becomes $T_c\propto k_{min}$.

Remarkably, similar arguments are still true in the mean-field approximation if we neglect degree correlations in the network and suppose in (11) $\zeta _{2}\simeq \langle {k}\rangle$, which leads to

12 $$\begin{aligned} T_c\simeq 2J\langle {k}\rangle . \end{aligned}$$

Equations (11), (12) play a crucial role in phase transition occurring in the finite size Ising model. The equations establish a clear connection between the system temperature and network statistical properties. For a given temperature, *T*, the critical value, $\zeta _{2,c}$, is determined from (11) as

13 $$\begin{aligned} \zeta _{2,c}=\frac{T}{2J}. \end{aligned}$$

Then, in Eq. (10) we expand the function $tanh(x)\approx x-\frac{1}{3}x^3$ with $x=\frac{\beta }{2}(4JS_zk+h)$. In this limit we can represent Eq. (10) in the form of

14 $$\begin{aligned} A S_z - B S_z^3 + \frac{h}{2T}=0, \end{aligned}$$

where coefficients *A* and *B* are defined as

15 $$\begin{aligned} A = \frac{\zeta _{2}}{\zeta _{2,c}}-1, \quad B =\frac{\zeta }{3} \frac{ \zeta _{2}^3 }{\zeta _{2,c}^3}. \end{aligned}$$

From (14) in the vicinity of critical point $\zeta _2=\zeta _{2,c}$ at $h=0$ we obtain

16 $$\begin{aligned} S_z=\left[ \frac{3}{\zeta _{c}} \left( \frac{\zeta _{2}}{\zeta _{2,c}}-1\right) \right] ^{1/2}. \end{aligned}$$

The properties of parameter $\zeta$ may be inferred from Fig. 2a. The magnitude of $\zeta$ is finite at $\gamma =1$ and reaches its maximum value at $\gamma _{max}\simeq \frac{1}{2}[ ln(N)+2-\sqrt{ln(N)[ln(N)-4]}]$, which implies $\gamma _{max}\approx 2.21$ for the networks with $N=1000$ nodes established in Figs. 1 and 2a, respectively.

Equation (16) establishes the second order phase transition from paramagnetic (PM) state ($S_z=0$) to ferromagnetic (FM) one ($S_z\ne 0$), which occurs if normalized degree correlation function obeys the $\zeta _{2} \ge \zeta _{2,c}$ condition. For social network systems, such a phase transition means transformation from disorder to some ordering state with opinion formation or voting. Hence, phase transition for the analysed Ising model appears only due to finite size effects, cf.^40,59,60,62^.

Remarkably, for power-law degree distribution networks parameter $\zeta _{2}$ is the function of number of nodes *N*. The dependence of $\zeta _{2}$ versus *N* for various $\gamma$ is shown in Fig. 2b. As clearly seen, $\zeta _{2}$ grows significantly within the anomalous domain of $\gamma$. Therefore, social networks, which possess a growing number of hubs (decreasing $\gamma$), promote the occurrence of some ordering state; it may be characterized in terms of definite social polarization, which is discussed in more detail below.

In the presence of non-vanishing external (pump) field *h* for $\zeta _{2} \rightarrow \zeta _{2,c}$ the order parameter may be obtained in the form

17 $$\begin{aligned} S_z\simeq \left( \frac{3h}{4J\zeta _c \zeta _{2,c}} \right) ^{1/3}\simeq \left( \frac{3h}{4J \langle {k}\rangle _c} \right) ^{1/3}. \end{aligned}$$

The right-hand part of the Eq. (17) is valid for large degree exponent $\gamma$ with the valid factorization of parameters $\zeta _{2}$ and $\zeta$. Notably, the main features of average degree $\langle {k}\rangle$ in this limit resembles $\zeta _{2}$, see Fig. 2.

### Non-equilibrium phase transition in solaser

#### Basic approach

In this section we examine the social lasing effect as a non-equilibrium phase transition that occur in social systems possessing some specific features. The model we discuss in this work may be formulated in a general form in terms of the quantum field theory apparatus.

For simplicity we consider *N* two-level systems (TLS), which comprise gain laser medium; each system is inherent to a well-distinguished ground ( $|g\rangle$ ) and excited ( $|e\rangle$ ) states possessing different energies $E_g$ and $E_e$, ($E_e>E_g$), respectively. We describe TLS at states $|e\rangle$ and $|g\rangle$ by bosonic annihilation and creation operators, $\hat{a}_j$ and $\hat{a}_j^{\dag }$ for ground state and $\hat{b}_j$ and $\hat{b}_j^{\dag }$ for excited state, respectively. We characterize the *j*th TLS by resonant frequency (energy) of transition $\omega _j=E_{e,j}-E_{g,j}$ ($j=1,2,...,N$); note that we use units where the Plank constant equal to 1.

**Figure 3:**
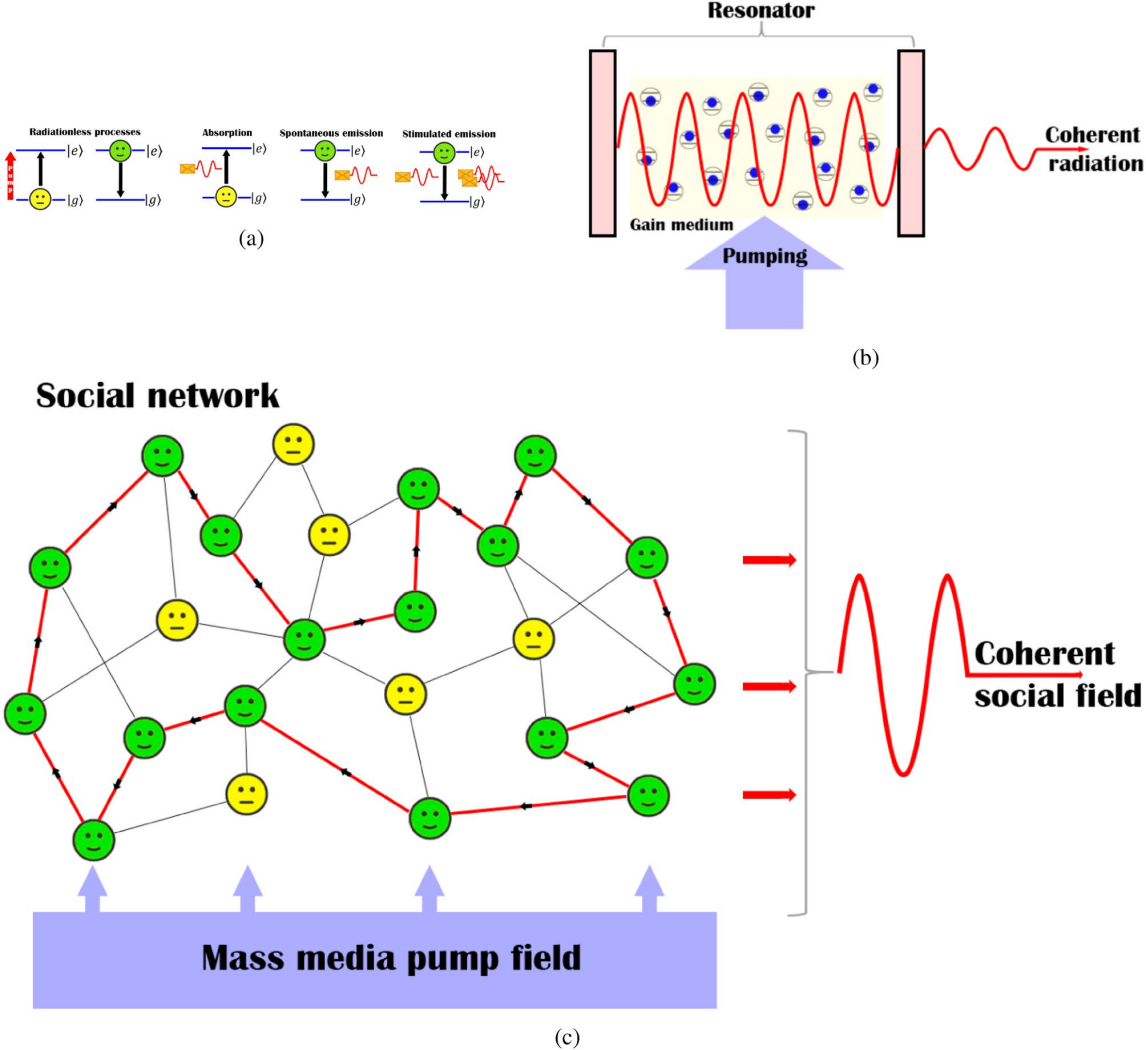
Sketch of (**a**) physical and (**b**) social laser systems, respectively. The processes, which are allowed in the solaser for TLS representing agent of DM, are shown in (**c**). The model of physical laser presumes interaction of resonator modes (only one mode is shown) with the ensemble of two level-oscillators (atoms) in the presence of an optical pump. At the core of solaser there is an echo chamber, which provides circulation and amplification of some privileged mode for the s-information field in a social network (illustrated with the red directed edges) in the presence of a strong social mass media pump. In (**c**) $|g\rangle$ and $|e\rangle$ are (emotionally) ground (neutral) and excited states, respectively.

We assume that interaction of TLSs ensemble with irradiation occurs in the resonator, which supports a multimode regime described by an annihilation (creation) field operator $\hat{f_v}, (\hat{f_v}^{\dag })$, which freely evolves in time with common frequency $\omega$, see Fig. 3a, cf.^77^. Within the framework of dipole and rotating-wave approximations, the generic Hamiltonian of the system is given by:

18 $$\begin{aligned} \begin{aligned} \hat{H}=-\frac{1}{2}\sum \limits _{j=1}^N \omega _{j}(\hat{b}_j^{\dag }\hat{b}_j-\hat{a}_j^{\dag }\hat{a}_j)+\frac{1}{N}\sum \limits _{j=1}^N\sum \limits _{v=1}^{k_j} \left[ \omega \hat{f_v}^{\dag }\hat{f_v}+ g_j(\hat{f_v}^{\dag }\hat{a}_j^{\dag }\hat{b}_j+\hat{b}_j^{\dag }\hat{a}_j \hat{f_v}) +iP(\hat{f_v}^{\dag }-f_v)\right] , \end{aligned} \end{aligned}$$

where $g_j$ characterizes interaction strength (energy) of TLS with field $\hat{f_v}$. Thereafter, we assume that $g_i=g$ for all TLS. In (18) $k_j$ is the *j*th node degree, which can be understood as a mean number of modes occurring in a physical laser resonator.

Physically, 2*g* represents so-called vacuum Rabi-splitting frequency, which indicates the rate, at which one quantum excitation may appear due to the interaction with a resonator field, cf.^37^. From quantum theory it is known that even in the absence of photons in a resonator (so-called vacuum state) the resonant probability of TLS transition from the ground state to the excited one within time *t* is $W=sin^2[gt]$, cf.^78^. Considering short time intervals such as $gt\ll1$ we can obtain $W\simeq g^2t^2$. Thereby, resonant excitation probability *W* of TLS is proportional to $g^2$.

In (18) *P* is an external strong classical field, which drives the gain medium. In physical laser the pump field is introduced into the resonator with the same frequency, $\omega$, which is inherent to a laser mode, see Fig. 3a.

In the framework of Heisenberg-Langevin formalism from (18) we obtain:

19a $$\begin{aligned}&\dot{\hat{f_v}}=(-i\omega -\kappa ) \hat{f_v}- ig \sum \limits _{j=1}^N \hat{p}_j +P(t) -i\hat{F}_{ph}, \end{aligned}$$

19b $$\begin{aligned}&\dot{\hat{p_j}}=(-i\omega _j-\Gamma _j)\hat{p}_j+ig\hat{S}_{zj}\sum \limits _ {v=1}^{k_j}\hat{f_v}-i\hat{F}_{1} \end{aligned}$$

19c $$\begin{aligned}&\dot{\hat{S_{zj}}}=\frac{1}{\tau _j}(\hat{S}_{zj,0}-\hat{S}_{zj})+2ig\sum \limits _{v=1}^{k_j}(\hat{f_v}^{\dag }\hat{p}_j-\hat{f_v} \hat{p}_i^{\dag })-\hat{F}_{2} \end{aligned}$$

where $\hat{p}_j=\hat{a}_j^{\dag }\hat{b}_j$ and $\hat{S}_{zj}=\hat{b}_j^{\dag }\hat{b}_j-\hat{a}_j^{\dag }\hat{a}_j$ define the *j*th TLS polarization (excitation) and levels population imbalance (inversion) operators, respectively; $\hat{S}_{zj,0}$ is a constant operator. For TLS, which establishes a DM agent features these operators may be recognized in the framework of quantum approach to DM theory, cf.^79,80^.

In Eq. (19) we describe the quantum model of driven-dissipative laser system in its general form; $\hat{F}_{ph},\hat{F}_{1,2}$ are standard Langevin quantum noise operators, which characterize laser medium coupling with the environment, cf.^7^.

The non-equilibrium features of the model given in Eq. (19) are characterized by parameters $\kappa$, $\Gamma _j$ and $\tau _j$. Physical lasers possess Fabry-Pérot (or some other) resonators, which in practice play a crucial role in formation and amplification of laser irradiation^77^. The medium, which possess some decay from resonator modes, is placed in the resonator Fig. 3a. The atoms composing the medium can absorb and emit near-resonant photons, which carry out some information. Without using a resonator, photons may be absorbed or lost after several acts of absorption and re-emission. With a resonator, laser field is reflected many times and gained before leaving the resonator. The losses of laser irradiation may appear, for example, due to non-ideal resonator mirrors. The resonator Q (quality)-factor for a given laser mode in this situation is determined by photon lifetime $\tau _{ph}\propto 1/\kappa$ in this mode, where $\kappa$ characterizes photon loss rate from the resonator^77^. Vanishing $\kappa$ corresponds to high Q-factor resonators, which prevent the leakage of laser field photons from the resonator. Roughly speaking, different resonator modes possess different values of $\kappa$. We neglect these differences in our model. We account non-equilibrium features of TLS in (19) by TLS depolarization (dephasing) rate $\Gamma _j$ and spontaneous emission characteristic time $\tau _j$, respectively.

#### The solaser model

Throughout this work we restrict ourselves by mean-field theory. In this case, we replace the operators in (19) by their mean values setting $p_j(t)=\langle \hat{p}_j\rangle$, $E(t)=\langle \hat{f_v}\rangle$, $\sigma ^{z}_j(t)=\langle {\hat{S}_{zj}}\rangle$ and $\langle {\hat{F}_{ph}}\rangle =\langle {\hat{F}_{1,2}}\rangle =0$ in average. As a result, from (19) we obtain semi-classical equations as following (cf.^8^)

20a $$\begin{aligned}&\dot{E}(t)=(-i\omega -\kappa )E(t) -ig \sum \limits _{j=1}^N p_j(t) +P(t), \end{aligned}$$

20b $$\begin{aligned}&\dot{p}_j(t)=(-i\omega _j-\Gamma _j)p_j(t) +i g k_j E(t) \sigma ^{z}_j(t), \end{aligned}$$

20c $$\begin{aligned}&\dot{\sigma }^{z}_j(t)=\frac{1}{\tau _j}(\sigma ^{z}_{j,0}-\sigma ^{z}_j(t))+ 2ig k_j (p_j(t)[E(t)]^*-[p_j(t)]^*E(t)). \end{aligned}$$

In (20) $\sigma ^{z}_j(t)$ describes the population imbalance for *j*th ($j=1,2,...,N$) TLS; $\sigma ^{z}_j(t)=1$ if two-level system occupies the upper $|e\rangle$ state, and $\sigma ^{z}_j(t)=-1$ if it occupies the lower one, $\sigma ^{z}_{j,0}$ is a constant value of population imbalance for *j*th TLS, which we specify below.

Let’s consider how the solaser system may be recognized in the framework of Eq. (20). Schematically, the solaser is shown in Fig. 3b.

At the core of solaser in Fig. 3b there is a social network system, which plays the role of the gain medium as it occurs with a usual physical laser device, cf. Fig. 3a. We propose so-called (social) s-atoms representing DM agents, which we model as a simple quantum-like TLS. For the solaser model we consider a ground state as emotionally neutral state. The “upper” state may be identified as emotionally excited (anger or happiness) state possessing some other social energy $E_e$. In Fig. 3c we summarize possible decisions of the agent in the framework of solaser paradigm. Such decisions are inherent to spreading or not, some socially actual information by a DM agent. In contrast to the routine Shannon’s information approach, here we discuss so-called s(social)-information related to some social news and events, which are meaningful only in terms of the problem for DM agents. The social field, *E*, possesses social energy, $\omega$, and acts as a carrier for s-information establishing social influence as a result. It is worth to notice that for some specific tasks in social and financial network systems we can interpret *E* as an information field which possess interference effects like a wave-function in quantum mechanics, cf.^52,80^.

Thus, we can recognize s-information spreading as a process of “absorption” and “emission” of s-quanta performed by spatially distributed DM agents similar to “common” two-level atoms, see Fig. 3c. Practically, we can consider these quanta of s-information as meaningful messages (like posts, tweets, retweets, emails, etc. ), containing some contextual information.

Emotional excitation of the agent emerges in two ways. Each act of s-photon absorption evokes probabilistic excitation of DM agent, which for simplicity possesses only two $|g\rangle$ and $|e\rangle$ quantum-like mental states, respectively, see Fig. 3c. On the other hand, an agent can change his/her state $|g\rangle$ to $|e\rangle$ under the mass media pump influence.

An agent in excited (emotional) state $|e\rangle$ can return back to its original (emotionally neutral ground) state in several ways as well. The transition of DM agent to state $|g\rangle$ occurs spontaneously, emitting s-photon (posting or publishing some tweet, for example). Stimulated transition to ground state $|g\rangle$ that is accompanied with emission of s-photon occurs in the presence of external s-photon, see Fig. 3c. The stimulated transitions, which occur in solaser coherently, are especially responsible for the amplification of information field. Spontaneous emission processes are incoherent; in physics they exhibit quantum nature of matter-light interaction^6–8^.

On the contrary, information amplification occurs due to (socially) stimulated emission process. As shown in Fig. 3c, stimulated emission presumes posting of a message stimulated by another. Remarkably, in this situation a DM agent can post spontaneous content, which reflects his/her feelings.

Roughly speaking, messages posted “spontaneously” may be distinguished from a “stimulated” one only by analysing the posting time. We expect socially stimulated posts of a DM agent to appear within the time when his/her followers are online. In this sense “stimulated” posts occur coherently as an immediate response to some discussions and posts appearing in social media. On the contrary, spontaneous emission occurs incoherently without any connection with some events in social media. Both of them depend on psychological state of DM agents. Moreover, in contrast to physical systems, socially stimulated posts may appear more “spontaneously” since they reflect some emotional variability of a DM agent for time moment of online posting. Remarkably, in this situation decision making appears under uncertainty and may be described in the framework of quantum approach to human cognition^79–83^. Human decisions occurring under uncertainty within limiting time clearly demonstrate quantum-like probabilistic features, cf.^84–89^. In this sense quantum approach to solaser, which we explore ab initio in this work, seems fully justified.

Notably, emotionally excited agents can change their mental state without any emission of s-photons. It may happen, for example, as a result of some discussions (“collisions”) with some other agents. The solaser quantum paradigm accounts all possible changes, which may occur with DM agent states $|e\rangle$ and $|g\rangle$ in the presence of s-information or without it.

#### Echo chambers as a solaser “resonator”

Without driving field *P* s-information disseminates in social media and may become lost after several (or, even many) steps, which are characterized by s-photon lifetime $\tau _{ph}\propto 1/\kappa$. To be more specific, for a network community we can define $\tau _{ph}$ as an average lifespan of a post. For well-known social media it varies from few minutes to several months, cf.^90,91^. For example, Twitter content changes very fast and message gets pushed down the page quickly.

Simultaneously, we can obtain (almost exponential) decrease of public attention to some events, for example, decreased attention to the Brexit and US presidential elections in 2016, cf.^92^. In Eq. (20) the *j*th TLS dephasing parameter $\Gamma _j$ is responsible for such a behaviour and characterizes potential attenuation of a DM agent’s attention to information field *E*.

The situation changes drastically in the presence of so-called echo chambers occurring with network communities, which possess absorption and re-emission of one type of contextual information for a given community, cf.^93^. Current socially oriented networks technically serve facilities on simultaneous s-information sharing with many users. For example, DM agents promote s-information spreading by posting, twitting, and re-twitting. The context of some meaningful information may be specified using hashtags. New facilities of social networks have generated principally new social phenomena with information dissemination, which are called echo chambers and homophily^94^. An echo chamber occurs in relatively close social networks, which possess selection and reinforcement of some contextual information (beliefs) due to information exchange and repetition inside the network, preventing it from other opinions, rebuttal, etc. The echo chambers in social network communities appear due to homophily feature of individuals, which support tendency to be connected together with similar people^95^. As a result, the echo chamber effect provides information induced selection (social polarization) of users (DM agents) who make their decisions being in good agreement with the information circulated in an echo chamber. Moreover, when DM agents encounter non-contextual (contradicting) information, they try to counteract this information and keep their beliefs. This is not surprising since famous social media environments like Twitter, Instagram, Facebook, Reddit, VK, Telegram, etc. contribute to formation of like-minded networks, unified by some key features and preferences for the content. In real world s-information spreading in the presence of echo chambers may be studied by examining topological properties of social networks and their specific peculiarities.

Remarkably, echo chambers occurring in current social media resemble laser resonators, which are able to enhance photon lifetime $\tau _{ph}$. We expect an echo chamber to sufficiently prolong s-information lifetime in social media as well, cf.^96^. In this regard we can introduce an echo chamber Q-factor parameter as cf.^37^

21 $$\begin{aligned} Q=\frac{\omega }{\kappa }=\frac{\omega \tau _{ph}}{2\pi }. \end{aligned}$$

In currently available quantum technologies high Q-factor resonators fulfill condition $Q\gg 1$^36^. The Q-factor for social echo chambers is a sophisticated question. We can suppose that high Q-factor echo chambers fulfill the condition $\omega \gg 2\pi /\tau _{ph}$. Clearly, such a condition may be achieved with some news representing keystone topics and remarkable events (like Brexit, Arab Spring, US elections, etc.) admitting frequent information re-circulation (in some form) and long time discussions. The discussions enable to examine steady state regimes of social behaviour. Homophily as an important social peculiarity may be associated here with the assumption that DM agents (TLS) interact with some common s-information field *E*, which is contextual (resonant) to the most part of DM agents within chosen social media (or networks), see (20). The interaction strength of a single TLS with *E* is characterized with *g*-parameter, see (20). Practically, we can recognize *g* as the rate, at which single DM agent polarization appears due to the interaction with echo chamber information field.

The tendency of DM agents to interact with only one type of s-information (which supports echo chamber) promotes a single mode regime when a social system produces one large macroscopic information field *E*^96^. This regime may also recognized as a information laser limit, cf.^52^.

### Solaser phase transition

We are starting with Ginzburg-Landau equation

22 $$\begin{aligned} \dot{E}= -\left( \kappa - \sum \limits _{j=1}^N\frac{(g^2 k_j \sigma ^{z}_{j,0} (\Gamma _j-i\Delta _j)}{\Delta _j^2+\Gamma _j^2}\right) E - \sum \limits _{j=1}^N \frac{4g^4 k_j^3 \tau _j \Gamma _j(\Gamma _j-i\Delta _j) \sigma ^{z}_{j,0}}{(\Delta _j^2+\Gamma _j^2)^2}|E|^2E +P, \end{aligned}$$

which governs solaser mean field *E* evolution in time (see section“Methods”). It is worth to notice that we restrict ourselves by third-order nonlinear term in Eq. (22) that is proportional to $|E|^2E$. The saturation effects which may lead to higher order nonlinearities in (22) are neglected.

Equation (22) admits significant simplification if we suppose that all TLSs in an echo chamber are intrinsically close to each other, i.e. we assume that conditions

23 $$\begin{aligned} \Gamma _j=\Gamma , \quad \tau _j=\tau , \quad \Delta _j\simeq 0 \end{aligned}$$

are fulfilled. The right-hand part of equation in (23) implies “strong” echo chamber effect when all DM agents are in complete resonance with circulated single mode information field *E*. The steady-state solution of (22) reads

24 $$\begin{aligned} \left( \frac{g^2 \langle {k}\rangle \sigma _{z} }{\Gamma } - \kappa \right) E - \frac{4g^4 {\langle {k}^3\rangle } \tau \sigma _{z}}{\Gamma ^2}E^3 +P=0, \end{aligned}$$

where $\sigma _z\equiv \sum \limits _{j}\sigma ^{z}_{j,0}=N\sigma ^{z}_0$ is macroscopic population imbalance; $\sigma ^{z}_0=\frac{1}{N}\sum \limits _{j} \sigma ^{z}_{j,0}$ is normalized population imbalance. The laser threshold is determined from (24) as

25 $$\begin{aligned} \sigma _{z,thr} =\frac{\kappa \Gamma }{g^2 \langle {k}\rangle }, \end{aligned}$$

or, for normalized population imbalance $\sigma ^{z}_0$ defined as

26 $$\begin{aligned} \sigma ^{z}_{0,thr}=\frac{\kappa \Gamma }{g^2 N \langle {k}\rangle }\equiv \frac{\kappa \Gamma }{[g(k)]^2 N}, \end{aligned}$$

where we made definition $g(k)\equiv g \sqrt{\langle {k}\rangle }$ which we explain latterly.

Equations (25), (26) represent one of the key result of this work. In particular, solaser gain medium implies dependence $\sigma _{z,thr}\propto {1/\langle k\rangle }$, which imply threshold-less laser phenomena ($\sigma _{thr}\rightarrow 0$) within anomalous domain $1<\gamma <2$. The combination of the parameters in denominator of (26) may be recognized as collective coupling parameter

27 $$\begin{aligned} G=g(k) \sqrt{N}=g\sqrt{N\langle {k}\rangle } \end{aligned}$$

for *N* two-level systems with complex network field *E*. In physics there exists $\sqrt{N}$ scaling law for matter-field interaction strength *g* if it occurs in gases, lattice environments, cf.^37^. However, as we can see from (27), the network environment can improve this scaling $\sqrt{\langle {k}\rangle }$ times. Since $\langle {k}\rangle$ may be large enough (see anomalous domain in Fig. 2a), collective parameter *G* admits significant improvement.

The macroscopic parameter of population imbalance $\sigma _z$ in laser theory plays a role of reciprocal temperature $\beta$, which is relevant to the whole system characterization for the Ising model^16^. Noteworthy, $\sigma _{z,thr}$ vanishes for large average node degree $\langle {k}\rangle$. In this sense the solaser represents a low-threshold laser system due to network peculiarities. On the other hand, for a given population imbalance, $\sigma _z$ Eq. (24), defines a threshold average node degree ${\langle {k}\rangle }_{thr}$ in the form

28 $$\begin{aligned} {\langle {k}\rangle }_{thr} =\frac{\kappa \Gamma }{g^2 \sigma _z }. \end{aligned}$$

Comparing (24) with (14), we can conclude that information field amplitude *E* (instead of $S_z$) represents the order parameter for dissipative phase transition to the laser (to be more specific, hereafter we are fixing the phase by considering real *E*)^16^. We rewrite (24) as

29 $$\begin{aligned} AE - B E^3 + P=0, \end{aligned}$$

where coefficients *A* and *B* are defined as

30 $$\begin{aligned} A = \left( \frac{\langle {k}\rangle }{{\langle {k}\rangle }_{thr}}-1 \right) \kappa , \quad B = \frac{4g^2\tau \kappa \zeta _{3}}{\Gamma }\frac{{\langle {k}\rangle }}{{\langle {k}\rangle }_{thr}}. \end{aligned}$$

Notably, coefficients *A*, *B* may be also obtained from laser rate-equations, cf.^12^. From (29) it is possible to infer that (phase) transition to lasing occurs at $A=0$.

In the vicinity of threshold point, i.e. for the networks, which possess $\langle {k}\rangle \rightarrow \langle {k}\rangle _{thr}$ from (29) at $P=0$ for the order parameter we get

31 $$\begin{aligned} E = \sqrt{\frac{A}{B}} =\sqrt{\frac{\Gamma }{4g^2 \tau \zeta _{3,thr}}\left( \frac{\langle {k}\rangle }{\langle {k}\rangle _{thr}}-1\right) }, \end{aligned}$$

Thus, above the threshold, i.e. for network nodes *N*, which admit condition $\langle {k}\rangle \ge \langle {k}\rangle _{thr}$ an establishment of finite (non-zero) information (social) field *E* occurs; it is accomplished with non-zero polarization (excitation) of TLS. We can recast order parameter *E* in the vicinity of $\langle {k}\rangle \simeq \langle {k}\rangle _{thr}$ through pumping *P* as

32 $$\begin{aligned} E= \left( \frac{P\Gamma }{4g^2\tau \kappa \zeta _{3,thr}}\right) ^{1/3}\simeq \left( \frac{P\Gamma }{4g^2\tau \kappa \langle {k}\rangle _{thr}^2}\right) ^{1/3}, \end{aligned}$$

where $\zeta _{3,thr}$ is normalized third order degree correlation parameter $\zeta _{3}$ taken at the threshold point, cf. (8). The right-hand part of the equation in (32) is valid for the networks for which $\zeta _{3}$ approaches $\langle {k}\rangle _{thr}^2$.

Equations (11–17) and (25–32) demonstrate an analogy between phase transitions occurring in the Ising model, which is considered by current social networks studies, and the solaser model, which we establish here, cf.^17^. In particular, an average node degree becomes a vital parameter for both of them within a random regime of complex networks where $\zeta _{2,3}$ admits factorization, i.e. for $\gamma >3$, see Fig. 2.

Dependence (32) for information field on social mass media pump *P* plays a crucial role in solaser features. In physics, gain medium pumped by strong classical field, which may be coherent or incoherent, see Fig. 3a. The latter possesses a broad spectrum^77^. In the framework of social studies the role of coherent mass-media pump is straightforward. It strongly supports echo chamber transition frequency $\omega$. The role of an incoherent social mass-media pump may be much more interesting, since in this case DM agents are influenced by strong and wide spectrum of different viewpoints. However, even in this case the social pumping effect may be strong enough due to so-called confirmation bias, which result from individuals’ feature to absorb information in a way that confirms one’s prior beliefs supported by an echo chamber. In social networks confirmation bias possess amplification by means of so-called filter bubbles; they display only the information that individuals are likely to agree with, while excluding opposing views^97^.

### Solaser dynamics and information diffusion

#### Viral information cascades in solaser

Modern social media creates unprecedented opportunities for DM agents to influence each other. This is facilitated by various Internet-based resources sharing applications (e.g. YouTube), blogs and microblogs (e.g. LiveJournal, Twitter etc.), social networks (e.g. Facebook, Myspace, VKontakte). In this sense, information field *E* responsible for the influence of DM agents cannot be considered as a constant in time, cf. (32).

For example, some information published by the user can almost immediately become “viral” and accessible to millions of people. It can provoke a rapid growth of so-called information cascades^98,99^. In this case, dissemination of information in social media is the object for close attention of researchers who work in the field of mathematics, computer science, statistical physics, sociology and psychology.

Time dependent phenomena for the solaser may be inferred from Ginzburg–Landau equation (22). In particular, from (22) for average s-photon number variable $n_{ph}=E^2$ (which corresponds to number of retweets, as example) we obtain

33 $$\begin{aligned} \dot{n}_{ph}=2A n_{ph} -2B n_{ph}^2 +2P(t)\sqrt{n_{ph}}, \end{aligned}$$

where we recover slow time dependence of pump $P\rightarrow P(t)$ on time.

**Figure 4:**
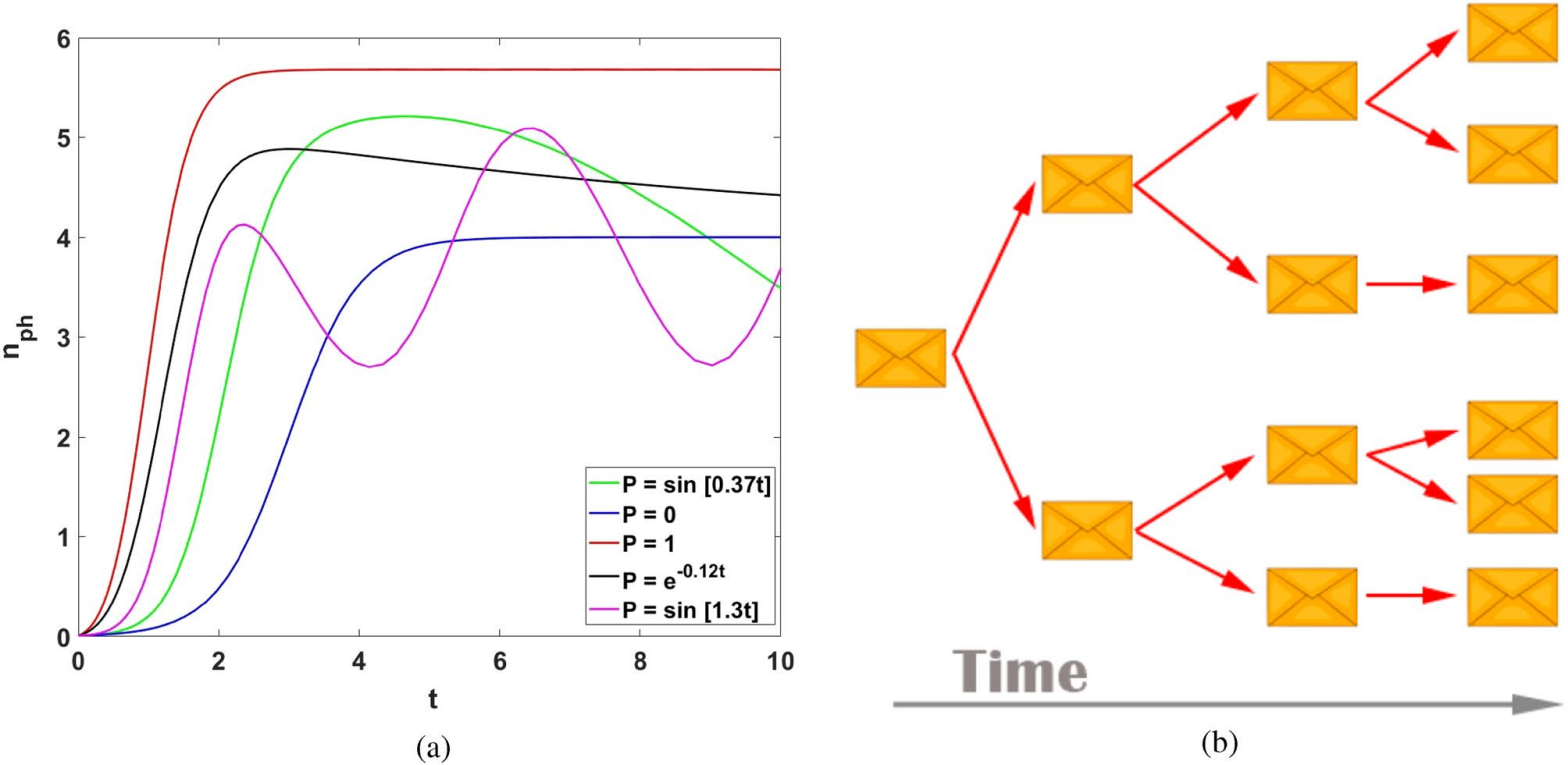
(**a**) Time dependence of average s-photon number $n_{ph}(t)$ for $A=1$ and $B=0.25$. Pump rates are: $P=0$ (blue curve), $P=1$ (red curve), $P(t)=sin(0.37t)$ (green curve), $P(t)=sin(1.3t)$ (magenda curve), and $P(t)=e^{-0.12t}$ (black curve), respectively. (**b**) Viral Information cascade in solaser network. The cascade starts with a socially stimulated repost (retweet) in the presence of spontaneously posted message, which together deliver information to two other agents in the network and initiate new “spontaneously” or socially stimulated messages. The messages occur in accordance with the rules explained in Fig. 3c.

Equation (33) may be recognized as a rate equation for the number of photons generated by laser at the output, cf.^8^. In the framework of social media studies, the similar equation (with $P=0$) characterizes a so-called susceptible-infected-susceptible (SIS) epidemic model, which describes information spread in social media based on the growth of infected agents population, cf.^44,66^. Recent remarkable applications of such a model to rumor and misinformation spreading are given in^100,101^.

In laser theory the pump field plays a crucial role. It is used to create required population inversion $\sigma _z$. Then, the pump may be switched-off, $P=0$. In this limit Eq. (33) admits a simple analytical solution

34 $$\begin{aligned} n_{ph}=\frac{A ve^{2At}}{1+B v e^{2At}}, \end{aligned}$$

where we define $v=\bar{n}/(A-B \bar{n})$; $\bar{n}$ is an average photon number at initial time $t=0$.

Figure 4a exhibits another important result of this work and explains the role of the mass media pump in the solaser. As seen in Fig. 4a, we plot solutions of Eq. (33) for s-photon average number $n_{ph}$ for various pump *P*(*t*). We can suppose that $n_{ph}$ describes reinforcement of some viral information supported by the echo chamber. Parameter $g^2\langle {k}\rangle \sigma _z/\Gamma$ in this case simply describes message diffusion rate whereas $\kappa$ characterizes recovery rate; the s-photons escape from the echo chamber, which amplifies this information with rate $\kappa$, cf. Eq. (24).

The blue curve in Fig.  4a is relevant to typical S-shape behavior, which is relevant to the SIS model and characterizes the (exponential) growth of $n_{ph}$ above the threshold ($A>0$). The pump field creates the solaser threshold and then switches off, $P=0$. This limit corresponds to growing information cascade, which is schematically depicted in Fig. 4b. In the solaser, a cascade starts with a “spontaneously” posted message. Then, in the presence of required inversion, or a relevant average node degree this message evokes avalanche of messages (s-photons) in the network created by other DM agents as a result of socially stimulated emission phenomena, cf. Fig. 3b.

In particular, such a cascade may be described in the framework of cascade generating function, which characterizes details of cascade microscopic dynamics, cf. patterns (3), (5) given in Fig. 2 of^102^. At the same time, the information cascade shown in Fig. 4b may be explained in the framework of structural virality paradigm, which considers viral information diffusion^103^.

In the presence of the pump of mass media an average s-photon number, as it is shown Fig. 4a, follows pump main features. In particular, a permanent pump possesses essential acceleration of information diffusion, see the red curve in Fig.  4a. For the black and magenta curves we suppose that $P(t)=P_0 sin[\nu t]$, where $P_0$ is a pump field amplitude, and $\nu$ is frequency of the pump variation. In adiabatic approximation ($\nu \ll \omega$) DM agents follows mass media pump evolution, which is clearly seen for the black and magenta curves behavior shown in Fig. 4a, respectively. Within the established time window, the green curve may be associated with power law decay of an information cascade. In contrast, the black line in Fig. 4a characterizes the exponential decay of the cascade demolished by mass media pump $P(t)=P_0 e^{-\nu _e t}$. Within the large time window, it approaches the blue curve obtained at $P=0$.

Thus, the plots in Fig. 4a may reproduce a variety of information cascade properties obtained experimentally depending on a mass media pump field action *P*(*t*), cf.^49,90,101,104,105^.

In particular, the SIS model in the framework of solaser possesses simple and very elegant interpretation. The DM agent is initially susceptible to s-photon. The s-photon absorption evokes infection of the agent with some probability, which corresponds to transition to the excited state. Then, s-photon emission (which practically implies posting a tweet, for example) changes the s-photon number (number of tweets) and simultaneously transfers DM agent to the ground state back. Notably, the same agent again becomes susceptible to absorb s-photon, which agrees with basic SIS model predictions^100,101^. Thus, the total number of emitted s-photons in the solaser model is accompanied with diminishing of population inversion. The lasing threshold in this case corresponds to the epidemic threshold.

If the lasing threshold does not fulfill ($A<0$), the properties of $n_{ph}$ strictly depends on the pump *P*. In particular, in this limit it is possible to obtain

35 $$\begin{aligned} n_{ph}=\frac{(e^{At}(A\sqrt{\bar{n}}+P)-P)^2}{A^2}, \end{aligned}$$

For $A<0$ Eq. (35) provides critical pump field $P_c=A^2 \bar{n}$ when $n_{ph}=\bar{n}$. For $P>P_c$ the $n_{ph}$ grows exponentially.

#### How fast s-information may be reinforced in solaser?

This is a keystone question that we can address to the solaser paradigm from the beginning. Up to date it is unclear how network peculiarities affect solaser gain medium, cf. Fig. 3b. As we have already pointed out, Fig. 4 and (34) may be understood in the framework of various models of disease spreading. To be more specific, even simple SI model can help to create a link between information growth presented in Fig. 4a and network peculiarities. To demonstrate that, let us examine the solaser model well above the threshold with a switched-off pump field, $P=0$ (we can simply neglect by $\kappa$ for the SI model in this limit). At any time moment we suppose that all DM agents in a social network belong to two large classes of “infected” or “susceptible” individuals. A DM agent belongs to set of “infected” population if he/she posts a message about some potentially viral content. Otherwise, he/she is “susceptible” (S). Notably, transfer from “infected” to “susceptible” agents within this model is not provided, which is clearly inherent to the SI approach.

We suppose that an “infected” agent posts the message, which is passed to a “susceptible” one (here we refer to so-called SI model for simplicity). Both of them are part of the solaser social network community, see Fig. 4b. Within the time interval *dt* a probability that corresponds to the message passage to a “susceptible” agent is $\epsilon d t$ (we suppose that $\epsilon$ is small enough, $\epsilon dt\ll 1$). Then, let us assume that “susceptible” agent *j* possesses node degree $k_j$. In this case a “susceptible” agent receives the message (becomes “infected”) with probability $\epsilon k_j dt$. The enhancement of this probability $k_j$ times is obvious: people more intensively communicated with individuals who possess more (communicative) links in social media. Let us assume that $k_j$ approaches its average value $\langle k \rangle$. Fraction of nodes with average degree *k*, which are “infected” in the network, is define as *I*, and the fraction, which are not “infected”, is defined as $1-I$, respectively. A simple equation that governs *I* variable is represented as (cf.^44^)

36 $$\begin{aligned} \dot{I}=\epsilon (1-I) \langle k \rangle I. \end{aligned}$$

The solution of Eq. (36) is

37 $$\begin{aligned} I=\frac{I_0e^{\epsilon \langle k \rangle t}}{1-I_0 + I_0 e^{\epsilon \langle k \rangle t}}, \end{aligned}$$

where $I(t=0)=I_0$ defines the initial condition.

Comparing Eq. (37) with (34) we can conclude that $\epsilon \simeq 2g^2 \sigma _{z}/\Gamma$.

Equation (37) provides a definition of characteristic time scale $t_k$ required to achieve 1/*e* fraction of all “susceptible” DM agents; from (37) we obtain

38 $$\begin{aligned} t_k=\frac{1}{\epsilon \langle k \rangle }\simeq \frac{1}{2A}, \end{aligned}$$

where we omit the term with $\kappa$, which is relevant to losses.

The obtained in (38) plays an important role for complex networks with the power-law degree distribution, which mimics a real life social media. Actually, the characteristic time scale $t_k$ is inversely proportional to the rate, at which a viral message spreads through social media. As it is follows from the inset to Fig. 2, social networks with degree exponent $\gamma >3$ may be recognized as socially passive in the framework of interaction exchange. The situation changes in the solaser limit. Echo chambers provide increasing of information exchange and social excitation of network agents. For the networks with power-law degree distribution it means vanishing degree exponent $\gamma$, which leads to clusterization and hubs appearing, cf. Fig. 1. The rate of information transfer enormously increases ($t_k$ vanishes) within anomalous network domain $1<\gamma <2$ which corresponds to growing $\langle k \rangle$. Notably, such an effect almost no longer depends on $\kappa$. This regime corresponds to a so-called superstrong coupling regime, when coupling between TLSs and resonator field behaves strongly enough during one round trip in the resonator, cf.^106^. Hence, we can conclude that in the framework of social laser paradigm we can expect huge acceleration of information spread and rapid information cascade, which result from a superstrong coupling regime. We can represent such a condition in the form

39 $$\begin{aligned} G \gg \Gamma , \quad \kappa ,\quad \omega _{FSR}, \end{aligned}$$

where $\omega _{FSR}$ is a free spectral range of the resonator, which is inversely proportional to the “size” of the resonator^106^.

In quantum physics, a strong coupling regime provides observation of various coherent matter-field interaction effects, like exciton-polariton Bose-Einstein condensates^32^. Roughly speaking, in this limit we need no population imbalance at all. It may be also seen from definition (25); threshold value of $\sigma _{thr}$ vanishes at $g(k)\gg \kappa , \Gamma$ for large *N*, cf. Fig. 2b.

We expect that it is possible to obtain condition (39) by choosing an appropriate network topology within $1<\gamma <2$ domain. This result represents an important impact for current social science. It means that in the presence of a strong mass media pump and echo chambers occurring in social media we do not need to obtain a large population inversion, which leads to large number of emotionally excited people. It is enough to have some strong majority who can attract people, create echo chambers in social networks and provide necessary network communicative topology (which characterised by some specific $\gamma$ for power-law networks, in particular). It is worth noticing that huge information cascades in this situation appear due to a spontaneous DM process. Then, all population is likely to follow these cascades.

## Methods

We may recognize social laser as a “usual” laser possessing complex network medium, which mimics a real-world social system and undergoes strong external information pressure. In this regard we assume that in the regime of solaser each of TLS adiabatically follows information field *E*, which evolves with frequency $\omega$. To be more specific, we consider solutions of Eq. (20) setting $E(t)=Ee^{-i\omega t}$, $P(t)=Pe^{-i\omega t}$ and $p_j(t)=p_je^{-i\omega t}$, which remove “fast” oscillations from Eq. (20). Setting $\dot{\sigma }^{z}_j(t)=0$ we assume that within new time scales population imbalance slowly varies with time. Then, from Eq. (20) we obtain

40a $$\begin{aligned}&\dot{E}=- \kappa E -ig \sum \limits _{j=1}^N p_j +P, \end{aligned}$$

40b $$\begin{aligned}&\dot{p}_j=-(i\Delta _j+\Gamma _j)p_j +i g k_j E \sigma ^{z}_j, \end{aligned}$$

40c $$\begin{aligned}&\sigma ^{z}_j=\sigma ^{z}_{j,0} +2i\tau _j g k_j (p_jE^* -p_j^*E), \end{aligned}$$

where $\Delta _j\equiv \omega _j-\omega$ is detuning from the resonant interaction of TLS with s-field. Detuning simply characterizes opinion diversity of a DM agent (described by frequency $\omega _j$) from the information mode (with frequency $\omega$) supported by echo chamber. From quantum theory it is known that the resonance condition implies $\Delta _j=0$, which corresponds to maximal excitation of TLS. For large values of $\Delta _j$ the probability of TLS excitation vanishes as $W\propto 1/((2g)^2+\Delta ^2)$, cf.^78^.

In the framework of social science $\Delta _j$ indicates how s-information (which is carried out by field *E*) is sensitive (relevant, contextual) to internal mental levels of the *j*th DM agent. In practice, $\Delta _j$ represents a stochastic (in time) variable obeying a distribution function. However, in the presence of strong echo chamber effect a social network system clearly exhibits extreme polarization of social community, which may be described by macroscopic population of two DM levels, cf.^107^. Thus, in this limit we expect to obtain $\Delta _j\simeq 0$ for all DM agents affiliated with a given echo chamber. We examine laser solution of Eq. (40), which is reminiscent to phase transition paradigm, cf.^8^. In the zeroth order approximation we can assume that $\sigma ^{z}_j\approx \sigma ^{z}_{j,0}$ (40) and $\dot{p}_j=0$, which imply

41 $$\begin{aligned} p_j\simeq \frac{i g k_j }{i\Delta _j+\Gamma _j}E \sigma ^{z}_{j,0}. \end{aligned}$$

Substituting Eqs. (41) into (40c) we obtain TLS population imbalance in the first order approximation as

42 $$\begin{aligned} \sigma ^{z}_j\simeq \sigma ^{z}_{j,0} -\frac{4g^2 k_j^2\tau _j \Gamma _j}{\Delta _j^2+\Gamma _j^2}\sigma ^{z}_{j,0}|E|^2, \end{aligned}$$

Thus, steady-state polarization of TLS obtained from (40b), reads

43 $$\begin{aligned} p_j\simeq \frac{i g k_j \sigma ^{z}_{j,0}}{i\Delta _j+\Gamma _j}\left( 1 - \frac{4g^2 k_j^2 \tau _j \Gamma _j}{\Delta _j^2+\Gamma _j^2}|E|^2\right) E. \end{aligned}$$

Equation (43) admit clear interpretation in the framework of current studies of echo chamber effect occurring in social media. As known, echo chambers promote polarization of social network communities shifting the entire group’s ideology to the extreme. Especially, it is clearly seen in social networks with political context, cf.^104,108^. Such a polarization exhibits strong bimodality of the distribution function for DM agents^96^. Moreover, due to psychological reasons DM agents work to shift their opinion within echo chamber realities. As it is follows from (43) the polarization is maximal for $\Delta _j\simeq 0$, i.e. in the limit of suppression of opinion diversity.

Substituting (43) into (40a) we arrive at complex Ginzburg–Landau equation (22).

## Discussion

In this work we offer the social laser concept to explain growing and fast-paced processes, which appear in current social media. The solaser, as any physical laser device, contains all keystone elements, which are necessary for reinforcement of social information. At the core of the solaser we propose social network media, which may be modeled (with some approximation) by means of networks whose degree distribution follows power law. The DM agents, which we established as simple quantum TLS, occupy the nodes of this network (we consider the case where each agent occupies only one node). An echo chamber, which mimics an optical resonator known from physical laser device, represents a key feature of social media. In particular, echo chamber may provide enhancement of one privileged s-information mode. We have demonstrated a clear link between the solaser and Ising model. The Ising model is used in sociophysics that considers networks and characterizes a social impact. Unlike the Ising model, the solaser accounts elementary processes of s-photon absorption, spontaneous emission and (socially) stimulated emission, which are relevant to on-line information exchange between DM agents in the network. As a result, social lasing represents a coherent nonequilibrium process that implies the formation of macroscopic social polarization, (viral) information cascade creation in the presence of population imbalance (social bias).

In this work, we restrict ourselves by mean-field theory, which has a twofold meaning for a solaser system. In particular, we obtain a set of equations to describe the solaser in mean-field approximation, which deals with average values of relevant operators and neglect all quantum (Langevin) noises. We examine a network structure in the limit when fluctuations of node degree may be neglected. In this case, features of the socially oriented Ising model and solaser provide the existence of some critical average node degree, which defines the second order phase transition threshold for the network topology and nodes.

In this limit we have shown that a coherent information field represents the order parameter and provides a huge social impact in solaser network media.

An important difference between the solaser and currently actual models of echo chambers and related phenomena is the presence of a mass media pump, which plays a crucial role in solaser generic features. The mass media pump provides population inversion required for lasing occurrence in the presence of various decay mechanisms. In this work we establish a clear link between the SIS/SI epidemiological models and the solaser rate equation for an average number of s-photons, which may be simply recognized as an average total number of messages posted or tweeted in a social network. We have shown that the social laser field immediately generates information cascades occurring in social media. Remarkably, the mass media pump enables to reinforce and accelerate cascade growth in different ways. Notably, DM agents follow an adiabatically time dependent mass media pump, which acts in a social network community and contributes to the reproduction of various reliable scenarios for information cascade evolution in time. We have achieved an important result, which relates to seminal features of parameter *g*(*k*) that characterizes coupling strength of DM agent with solaser s-information modes; we have shown that *g*(*k*) is proportional to $\sqrt{\langle k\rangle }$ for the network structure possessing average node degree $\langle k\rangle$. We have shown that the time of information spreading in a social network is inversely proportional to average node degree $\langle k\rangle$, which may be large enough in solaser systems. As a result, we can obtain a huge information cascade and social impact at the “output” of the solaser, which is manipulated by the mass media pump.

Let us briefly discuss some important and open problems, which arise in the framework of the proposed solaser paradigm.

First, it is important to elucidate the role of quantum processes occurring with the solaser at microscopic level, i.e. beyond mean-field theory. In particular, from laser theory it is known that quantum effects are important in the vicinity of threshold point, cf.^6–10^. In the framework of social science, quantum-like effects may be also recognized as some interference phenomena appearing in the Internet communities and can be explored for evaluation of indirect influence in social media, cf.^109^. In this sense, it is interesting to consider DM agents possessing three and more mental levels of decisions (or, emotions). Usual physical atoms interacting with e.m. fields in this case exhibit lasing phenomena due to constructive quantum interference of different quantum levels^78^.

Second, we can account network peculiarities in quantum-like DM problem where agents behave within some network environment. In particular, such an environment may be relevant to network structuring reservoir which we can account as uncertainty factor for DM agents, cf.^79^. In this case our approach may be useful tool for the problem of two- (or more) traders market in the presence of information exchange within some network, cf.^110^.

Third, it is interesting to consider quantum (statistical) theory for network degree correlations. We speak here about accounting of a network assortativity parameter, which allows to examine some vital characteristics inherent to real life social networks, cf.^70^. In this sense the networks model with power-law distribution of degree-degree distance seems to be more suitable for real-world networks characterization^73^. We are confident that the current work opens a new avenue in such studies.

## References

[1] Lamb WE, Schleich WP, Scully MO, Townes CH (1999). Laser physics: Quantum controversy in action. Rev. Mod. Phys..

[2] Bagaev S (2010). Beginning of the laser era in the ussr (collected papers).

[3] Milonni PW, Eberly JH (2010). Laser Resonators and Gaussian Beams.

[4] Blood P (2015). Quantum Confined Laser Devices: Optical gain and recombination in semiconductors.

[5] Robins NP, Altin PA, Debs JE, Close JD (2013). Atom lasers: Production, properties and prospects for precision inertial measurement. Phys. Rep..

[6] Scully MO, Lamb WE (1967). Quantum theory of an optical maser. i. general theory. Phys. Rev..

[7] Lax, M. Fluctuation and coherence phenomena in classical and quantum physics. *Part of Proceedings, 9th Brandeis University Summer Institute in Theoretical Physics : Statistical physics, phase transitions and superfluidity* (1968).

[8] Haken H (1985). Laser Light Dynamics.

[9] Glauber RJ (2007). Quantum Theory of Optical Coherence: Selected Papers and Lectures.

[10] Arecchi FT, Berné A, Bulamacchi P (1966). High-order fluctuations in a single-mode laser field. Phys. Rev. Lett..

[11] Haken H, Risken H, Weidlich W (1967). Quantum mechanical solutions of the laser masterequation. Z. Angew. Phys..

[12] Sargent M, Scully MO, Lamb WE (1970). Buildup of laser oscillations from quantum noise. Appl. Opt..

[13] Rice PR, Carmichael HJ (1994). Photon statistics of a cavity-qed laser: A comment on the laser-phase-transition analogy. Phys. Rev. A.

[14] Chaikin PM, Lubensky TC, Witten TA (1995). Principles of Condensed Matter Physics.

[15] Lifshitz EM, Pitaevskii LP (2013). Statistical Physics: Theory of the Condensed State.

[16] Graham R, Haken H (1970). Laserlight-first example of a second-order phase transition far away from thermal equilibrium. Z. Angew. Phys..

[17] DeGiorgio V, Scully MO (1970). Analogy between the laser threshold region and a second-order phase transition. Phys. Rev. A.

[18] Kessler EM, Giedke G, Imamoglu A, Yelin SF, Lukin JI, Cirac MD (2012). Dissipative phase transition in a central spin system. Phys. Rev. A.

[19] Zel’Dovich YB, Levich EV (1969). Bose condensation and shock waves in photon spectra. Sov. Phys. JETP.

[20] Hepp K, Lieb EH (1973). On the superradiant phase transition for molecules in a quantized radiation field: the dicke maser model. Ann. Phys..

[21] Wang YK, Hioe FT (1973). Phase transition in the dicke model of superradiance. Phys. Rev. A.

[22] Herrmann F, Wurfel P (2005). Light with nonzero chemical potential. Am. J. Phys..

[23] Herrmann F, Wurfel P (2001). The elusive chemical potential. Am. J. Phys..

[24] Leff HS (2015). Fluctuations in particle number for a photon gas. Am. J. Phys..

[25] Kocharovsky VV, Scully MO, Zhu SY, Zubairy MS (2000). Condensation of n bosons. ii. nonequilibrium analysis of an ideal bose gas and the laser phase-transition analogy. Phys. Rev. A.

[26] Snoke D (2012). Polariton condensation and lasing. Exciton Polaritons in Microcavities.

[27] Chestnov IY, Alodjants AP, Arakelian SM (2013). Lasing and high-temperature phase transitions in atomic systems with dressed-state polaritons. Phys. Rev. A.

[28] Kruchkov A, Slyusarenko Y (2013). Bose-einstein condensation of photons in an ideal atomic gas. Phys. Rev. A.

[29] Kirton P, Keeling J (2013). Nonequilibrium model of photon condensation. Phys. Rev. Lett..

[30] Sobyanin DN (2013). Bose–Einstein condensation of light: General theory. Phys. Rev. E.

[31] Ketterle W (2002). Nobel lecture: When atoms behave as waves: Bose-einstein condensation and the atom laser. Rev. Mod. Phys..

[32] Deng H, Haug H, Yamamoto Y (2010). Exciton-polariton bose-einstein condensation. Rev. Mod. Phys..

[33] Klaers J, Schmitt J, Vewinger F, Weitz M (2010). Bose–Einstein condensation of photons in an optical microcavity. Nature.

[34] Demokritov VE, Demidov V (2006). Bose–Einstein condensation of quasi-equilibrium magnons at room temperature under pumping. Nature.

[35] Zhang Z, Agarwal GS, Scully MO (2019). Quantum fluctuations in the fröhlich condensate of molecular vibrations driven far from equilibrium. Phys. Rev. Lett..

[36] Vahala KJ (2003). Optical microcavities. Nature.

[37] Miller R (2005). Trapped atoms in cavity qed: Coupling quantized light and matter. J. Phys. B: At. Mol. Opt. Phys..

[38] Bianconi G, Barabási AL (2001). Bose–Einstein condensation in complex networks. Phys. Rev. Lett..

[39] Park J, Newman ME (2004). Statistical mechanics of networks. Phys. Rev. E.

[40] Dorogovtsev SN, Goltsev AV, Mendes JF (2008). Critical phenomena in complex networks. Rev. Mod. Phys..

[41] Parisi G (1992). An introduction to the statistical mechanics of amorphous systems. Field Theory Disord. Simul..

[42] Schmidt E, Cohen J (2013). The New Digital Age: Reshaping the Future of People, Nations and Business.

[43] Newman ME, Girvan M (2004). Finding and evaluating community structure in networks. Phys. Rev. E.

[44] Barabási, A. L.* Network Science.* (Cambridge University Press, 2016).

[45] Holyst JA (2016). Cyberemotions: Collective Emotions in Cyberspace.

[46] Cheng, J., Adamic, L., Dow, P. A., Kleinberg, J. M. & Leskovec, J. Can cascades be predicted? *Proceedings of the 23rd international conference on World wide web* 925–936 (2014).

[47] Krastev, I. In mistrust we trust: Can democracy survive when we don’t trust our leaders? *TED Conferences* (2013).

[48] Denselow J (2012). Why it’s still kicking off everywhere: The new global revolutions. Int. Affairs (Lond.).

[49] Hemsley J (2016). Studying the viral growth of a connective action network using information event signatures. First Monday.

[50] Khrennikov A (2016). Social laser: Action amplification by stimulated emission of social energy. Philos. Trans. R. Soc. A Math. Phys. Eng. Sci..

[51] Khrennikov AY (2020). Social Laser.

[52] Khrennikov A, Toffano Z, Dubois F (2019). Concept of information laser: From quantum theory to behavioural dynamics. Eur. Phys. J. Sp. Top..

[53] Khrennikov A, Alodjants A, Trofimova A, Tsarev D (2018). On interpretational questions for quantum-like modeling of social lasing. Entropy.

[54] Baumann F, Lorenz-Spreen P, Sokolov IM, Starnini M (2020). Modeling echo chambers and polarization dynamics in social networks. Phys. Rev. Lett..

[55] Cinelli M, Morales GDF, Galeazzi A, Quattrociocchi W, Starnini M (2021). The echo chamber effect on social media. Proc. Natl. Acad. Sci..

[56] Tsarev D, Trofimova A, Alodjants A, Khrennikov A (2019). Phase transitions, collective emotions and decision-making problem in heterogeneous social systems. Sci. Rep..

[57] Weidlich W (1973). Fokker-Planck Equation Treatment of Interacting Social Groups.

[58] Pastor-Satorras R, Vespignani A (2004). Evolution and Structure of the Internet: A Statistical Physics Approach.

[59] Weidlich W (2002). Mean field solution of the ising model on a barabási-albert network. Phys. Lett. A.

[60] Lee SH, Ha M, Jeong H, Noh JD, Park H (2009). Critical behavior of the ising model in annealed scale-free networks. Phys. Rev. E.

[61] Stauffer D (2008). Social applications of two-dimensional ising models. Am. J. Phys..

[62] Holovatch Y (2017). Order, Disorder And Criticality-Advanced Problems Of Phase Transition Theory.

[63] García-Diaz C (2013). Serge galam: Sociophysics: A physicist’s modeling of psycho-political phenomena. J. Artif. Soc. Soc. Simul..

[64] Kohring GA (1996). Ising models of social impact: the role of cumulative advantage. J. Phys..

[65] Holyst JA, Kacperski K, Schweitzer F (2000). Phase transitions in social impact models of opinion formation. Phys. A.

[66] Pastor-Satorras R, Castellano C, Van Mieghem P, Vespignani A (2015). Epidemic processes in complex networks. Rev. Mod. Phys..

[67] Ostilli M (2010). Statistical mechanics of rumour spreading in network communities. Proc. Comput. Sci..

[68] Mello IF, Squillante L, Gomes GO, Seridonio AC, de Souza M (2021). Epidemics, the ising-model and percolation theory: A comprehensive review focused on COVID-19. Phys. A.

[69] Bazhenov AY, Tsarev DV, Alodjants AP (2021). Mean-field theory of superradiant phase transition in complex networks. Phys. Rev. E.

[70] Guleva V (2020). Emerging complexity in distributed intelligent systems. Entropy.

[71] Vitiello G (1995). Dissipation and memory capacity in the quantum brain model. Int. J. Mod. Phys. B.

[72] Vitiello, G. *My double unveiled: The dissipative quantum model of brain* (Advances in Consciousness Research, John Benjamins Publishing Company, 2001).

[73] Zhoua B, Meng X, Stanley E (2020). Power-law distribution of degree-degree distance: A better representation of the scale-free property of complex networks. PNAS.

[74] Dragulescu A, Yakovenko VM (2000). Statistical mechanics of money. Eur. Phys. J. B Condens. Matter Complex Syst..

[75] Chakraborti A, Chakrabarti BK (2000). Statistical mechanics of money: How saving propensity affects its distribution. Eur. Phys. J. B Condens. Matter Complex Syst..

[76] Mimkes J (2006). A thermodynamic formulation of social science. Econophys. Sociophys..

[77] Svelto O, Hanna DC (1998). Principles of Lasers.

[78] Scully MS, Zubairy M (1997). Quantum Optics.

[79] Bagarello F, Basieva I, Khrennikov A (2018). Quantum field inspired model of decision making: Asymptotic stabilization of belief state via interaction with surrounding mental environment. J. Math. Psychol..

[80] Asano M, Basieva I, Khrennikov A, Ohya M, Tanaka Y (2012). Quantum-like dynamics of decision-making. Phys. A.

[81] Khrennikov A (2010). Ubiquitous Quantum Structure: From Psychology to Finances.

[82] Busemeyer JR, Bruza PD (2012). Quantum Models of Cognition and Decision.

[83] Haven E, Khrennikov AY, Robinson TR (2017). Quantum Methods in Social Science: A First Course.

[84] Busemeyer JR, Wang Z (2015). What is quantum cognition, and how is it applied to psychology?. Curr. Dir. Psychol. Sci..

[85] Plotnitsky A (2014). Are quantum-mechanical-like models possible, or necessary, outside quantum physics?. Phys. Scr..

[86] Surov IA, Pilkevich SV, Alodjants AP, Khmelevsky SV (2019). Quantum phase stability in human cognition. Front. Psychol..

[87] Wichert A, Moreira C, Bruza P (2020). Balanced quantum-like bayesian networks. Front. Psychol..

[88] Lawless W (2020). Quantum-like interdependence theory advances autonomous human–machine teams (a-hmts). Entropy.

[89] Tonello L, Grigolini P (2021). Approaching bounded rationality: From quantum probability to criticality. Entropy.

[90] Quattrociocchi, W., Scala, A. & Sunstein, C. R. Echo chambers on facebook. *SSRN* 2795110 (2016).

[91] Symonds, J. How long do they last? https://the-refinery.io/blog/how-long-does-a-social-media-post-last. (2021).

[92] Mikhailov AP, Petrov AP, Pronchev GB, Proncheva OG (2018). Modeling a decrease in public attention to a past one-time political event. Doklady Math..

[93] Khrennikov A (2020). Social laser model for the bandwagon effect: Generation of coherent information waves. Entropy.

[94] Jamieson KH, Cappella JN (2008). Echo chamber: Rush Limbaugh and the conservative media establishment.

[95] McPherson M, Smith-Lovin L, Cook JM (2001). Birds of a feather: Homophily in social networks. Ann. Rev. Sociol..

[96] Bessi A (2016). Homophily and polarization in the age of misinformation. Eur. Phys. J. Sp. Top..

[97] Kitchens B, Johnson SL, Gray P (2020). Understanding echo chambers and filter bubbles: The impact of social media on diversification and partisan shifts in news consumption. MIS Quart..

[98] Watts DJ (2002). A simple model of global cascades on random networks. Proc. Natl. Acad. Sci..

[99] Chen W, Lakshmanan LV, Castillo C (2013). Information and influence propagation in social networkss. Synth. Lect. Data Manag..

[100] Maleki, M., Mead, E., Arani, M. & Agarwal, N. Using an epidemiological model to study the spread of misinformation during the black lives matter movement. arXiv preprint arXiv:2103.12191 (2021).

[101] Jin, F., Dougherty, E., Saraf, P., Cao, Y. & Ramakrishnan, N. Epidemiological modeling of news and rumors on twitter. *Proceedings of the 7th workshop on social network mining and analysis* 1–9 (2013).

[102] Ghosh, R. & Lerman, K. A framework for quantitative analysis of cascades on networks. *In Proceedings of the fourth ACM international conference on Web search and data mining* 665–674 (2011).

[103] Goel S, Anderson A, Hofman J, Watts DJ (2016). The structural virality of online diffusion. Manag. Sci..

[104] Cinelli M, Morales GD, Galeazzi A, Quattrociocchi W, Starnini M (2021). The echo chamber effect on social media. Proc. Natl. Acad. Sci..

[105] Matsubara, Y., Sakurai, Y., Prakash, B. A., Li, L. & Faloutsos, C. Rise and fall patterns of information diffusion: model and implications. *Proceedings of the 18th ACM SIGKDD international conference on Knowledge discovery and data mining* 6–14 (2012).

[106] Meiser D, Meystre P (2006). Superstrong coupling regime of cavity quantum electrodynamics. Phys. Rev. A.

[107] Sasahara K (2021). Social influence and unfollowing accelerate the emergence of echo chambers. J. Comput. Soc. Sci..

[108] Becker J, Porter E, Centola D (2019). The wisdom of partisan crowds. Proc. Natl. Acad. Sci..

[109] Shuai X (2012). Modeling indirect influence on twitter. Int. J. Semant. Web Inf. Syst..

[110] Bagarello F, Haven E (2014). The role of information in a two-traders market. Phys. A: Stat. Mech. its Appl..

